# The INIS Study. International Neonatal Immunotherapy Study: non-specific intravenous immunoglobulin therapy for suspected or proven neonatal sepsis: an international, placebo controlled, multicentre randomised trial

**DOI:** 10.1186/1471-2393-8-52

**Published:** 2008-12-08

**Authors:** The INIS Study Collaborative Group

**Affiliations:** 1 National Perinatal Epidemiology Unit

## Abstract

**Background:**

Sepsis is an important cause of neonatal death and perinatal brain damage, particularly in preterm infants. While effective antibiotic treatment is essential treatment for sepsis, resistance to antibiotics is increasing. Adjuvant therapies, such as intravenous immunoglobulin, therefore offer an important additional strategy. Three Cochrane systematic reviews of randomised controlled trials in nearly 6,000 patients suggest that non-specific, polyclonal intravenous immunoglobulin is safe and reduces sepsis by about 15% when used as prophylaxis but does not reduce mortality in this situation. When intravenous immunoglobulin is used in the acute treatment of neonatal sepsis, however, there is a suggestion that it may reduce mortality by 45%. However, the existing trials of treatment were small and lacked long-term follow-up data.

This study will assess reliably whether treatment of neonatal sepsis with intravenous immunoglobulin reduces mortality and adverse neuro-developmental outcome.

**Methods and design:**

A randomised, placebo controlled, double blind trial. Babies with suspected or proven neonatal sepsis will be randomised to receive intravenous immunoglobulin therapy or placebo.

Eligibility criteria

Babies must be receiving antibiotics and have proven or suspected serious infection AND have at least one of the following: birthweight less than 1500 g OR evidence of infection in blood culture, cerebrospinal fluid or usually sterile body fluid OR be receiving respiratory support via an endotracheal tube AND there is substantial uncertainty that intravenous immunoglobulin is indicated.

Exclusion criteria

Babies are excluded if intravenous immunoglobulin has already been given OR intravenous immunoglobulin is thought to be needed OR contra-indicated.

Trial treatment

Babies will be given either 10 ml/kg of intravenous immunoglobulin or identical placebo solution over 4–6 hours, repeated 48 hours later.

Primary outcome

Mortality or major disability at two years, corrected for gestational age.

Data collection

Data will be collected at discharge from hospital and at 2 years of age (corrected for gestation) using a parental questionnaire and a health status questionnaire completed during a face-to-face follow-up appointment with the child's paediatrician.

**Trial registration:**

Current Controlled Trials ISCRTN94984750.

## Background

This protocol is for a large, simple-in-design, double blind, placebo controlled, pragmatic, multicentre randomised trial.

### Hypothesis to be tested

That, in infants receiving antibiotics for clinical sepsis, the addition of non-specific, polyclonal intravenous immunoglobulin IgG (IVIG) therapy reduces mortality and major morbidity compared with antibiotics alone.

### Background

Neonatal sepsis is a major cause of mortality and morbidity and has been implicated in the causation of perinatal brain damage and cerebral palsy, both in term and preterm infants [1,2]. Although antibiotics are the mainstay of therapy, increasing numbers of bacteria are resistant to them [3,4]. Effective adjunctive strategies are therefore needed.

### Incidence, potential impact on mortality and problems in diagnosis

In a prospective study in seven Australian neonatal intensive care units (NICUs), Isaacs and colleagues reported an annual incidence of sepsis of 6.6 per 1000 live births, of which 75% were late onset (more than 48 hours after birth). Overall hospital mortality for sepsis was 10% [5]. In a cohort of 54 UK neonatal units in 1998, 204 (5%) of 3,963 consecutive admissions to neonatal units had a positive blood culture [6]. Of these, 16 (8%) died. Of 3,759 (95%) babies with negative blood cultures, 95 babies died (2.5%). For very low birthweight (VLBW) infants with positive blood cultures, mortality was 14%. In a North American cohort, mortality in VLBW infants with septicaemia was 21% [7]. However, these figures may underestimate the true incidence of neonatal sepsis. Blood cultures may often be negative if less than 1 ml of blood is sampled [8]. Furthermore, while sepsis was the primary cause of death in most infants under 1000 g at autopsy, it was clinically undiagnosed in 61% of cases [9]. Sepsis-specific mortality rates should therefore be interpreted with caution, as the diagnosis may often be inaccurate. More reliable evidence would be provided by randomised comparisons of the effects of specific interventions on mortality from all causes.

### Potential impact of sepsis on the perinatal brain

Recent evidence suggests that sepsis is also important in the pathogenesis of neuro-developmental impairment of perinatal origin. In a case-control study of 424 births, Grether and Nelson found an association between maternal infection in labour and cerebral palsy in infants with birthweight of at least 2500 g (OR 9.3, 95% CI 3.7, 23.0).

In another case-control study of 96 term infants, levels of cytokines in neonatal blood spots were consistently higher in children diagnosed with cerebral palsy at 3 years of age than in controls, suggesting that an inflammatory response may be important in the aetiology of cerebral impairment [10]. In preterm infants, sepsis is also associated with subsequent adverse neuro-developmental outcome [2]. Dammann and Leviton have suggested that infection remote from the preterm brain may predispose to cerebral white matter damage with disruption of oligodendroglial myelination and disordered migration of precursors [11]. The damage could result partly from inadequate endogenous protection from developmentally regulated factors such as oligotrophins [12]. As antenatal and postnatal sepsis may predispose to neuro-developmental impairment and disability in term and preterm infants, these are essential measures of outcome.

### Possible adjunctive treatments

#### Immunoglobulin

Newborn infants, particularly those who are very low birthweight or preterm, are deficient in IgG, which binds to cell surface receptors, provides opsonic activity, activates fixation of complement, promotes antibody dependent cytotoxicity, improves neutrophil chemiluminescence and phagocytosis and can improve neutropenia by enhancing the release of stored neutrophils [13-16]. Intravenous immunoglobulin (IVIG) is therefore a theoretically attractive strategy, with multiple mechanisms of action. Its potential clinical relevance is confirmed by recent evidence from randomised controlled trials (see below).

#### Pentoxifylline

In animal models of sepsis, pentoxifylline, a methylxanthine deriviative, inhibits production of Tumor Necrosis Factor (TNF), preserves micro-vascular blood flow, prevents circulatory failure and intestinal vaso-constriction and improves survival [17,18]. It is well tolerated and decreases TNF production in adults and preterm infants with sepsis [19-21]. Two randomised controlled trials (RCTs) of pentoxifylline recruited 140 preterm infants with clinical sepsis [21,22]. Among the 107 with positive blood cultures, pentoxifylline was associated with an 86% reduction in risk of mortality (RR 0.14, 95% CI 0.03, 0.76). Outcomes for the 33 infants not included in the analyses are not available. Pentoxifylline may be a promising therapy in neonatal sepsis.

#### Cytokines

Other adjunctive strategies for prophylaxis or treatment of neonatal sepsis are also attractive, such as use of the recombinant cytokines Granulocyte Colony Stimulating Factor (G-CSF) or Granulocyte-Macrophage Colony Stimulating Factor (GM-CSF) to prevent neutropenia [23]. However, no systematic reviews of RCTs of these agents are yet available. In four RCTs of G-CSF therapy which recruited 125 infants with neonatal sepsis, there was a trend to reduced mortality which was not statistically significant (OR 0.6, 95% CI 0.2,1.8) [24-27].

Two recent RCTs of GM-CSF prophylaxis in a total of 339 high risk infants showed no reduction in sepsis or mortality [28,29]. However, these findings do not rule out a moderate benefit [30].

#### Blood products other than immunoglobulin

White cell (granulocyte) transfusions are also a logical approach. Although preliminary clinical evidence is encouraging, there are potential risks from transmission of infection (e.g. HIV or hepatitis) or from graft-versus-host disease, and the technology is not widely available [31]. Exchange transfusion with fresh whole adult blood appeared effective in one RCT of 22 septicaemic infants, but may also transmit infection [32]. In another RCT, in 776 infants of less than 32 weeks' gestation, there was no evidence that prophylactic fresh frozen plasma reduced the risks of mortality from all causes or of disability in survivors at 2 years [33].

Overall, therefore, the evidence suggests that IVIG therapy is one of the most promising strategies in neonatal sepsis and should be assessed in a definitive RCT.

### Non-specific versus specific immunoglobulin

This trial will use non-specific, polyclonal IVIG (normal human IgG immunoglobulin) produced from plasma from non-UK donors. It was decided that specific IVIG would not be used and that there was no necessity to characterise the specific antibacterial profile of the non-specific IVIG for the following reasons:

1) Previous RCTs of non-specific, polyclonal IVIG in neonates and adults did not characterise any specific aspects of antibacterial function in the products used. There is therefore no reference laboratory data against which to judge the possible antibacterial efficacy of polyclonal IVIG.

2) As the mechanism of action of IVIG is likely to be multifactorial, the precise aspects of antibacterial function which should be assessed are speculative.

3) Despite the production of monoclonal antibodies with demonstrable in vitro and in vivo antibacterial function in laboratory studies, they have not been associated with reductions in mortality in RCTs. There is therefore no evidence that laboratory studies which attempt to characterise specific aspects of antibacterial function in IVIG products would be more predictive of clinical efficacy than the existing clinical evidence from RCTs in support of non-specific, polyclonal IVIG therapy.

### Results of previous randomised controlled trials

A Cochrane systematic review of the prophylactic use of non-specific IVIG in 15 RCTs with a total of 5,054 preterm or low birthweight infants has demonstrated that prophylactic, non-specific IVIG reduced sepsis (RR 0.85, 95% CI 0.74, 0.98) and was safe, with no major adverse effects, but did not demonstrate a definite reduction in mortality (RR 0.89, 95% CI 0.75, 1.05) [34].

A Cochrane systematic review of reports of RCTs of IVIG therapy for proven or suspected neonatal sepsis identified nine studies that reported outcomes for 318 infants with suspected infection and 262 infants with proven infection [15,35-43]. IVIG therapy appeared to be safe and was associated with approximately 40% reduction in the risk of mortality for both suspected and proven infection (Tables 1 and 2). However, the confidence intervals were wide and the studies included in the analyses were small and not of high methodological quality. Problems identified by the reviewers were lack of allocation concealment, lack of blinding of outcome assessment and high levels of post-randomisation exclusions in some of the trials.

**Table 1:** Mortality in trials of IVIG for suspected infection in neonates

**Study**	**Exptl n/N**	**Ctrl n/N**	**Relative Risk (95% CI Fixed)**	**Weight**
Christensen 1991	0/11	0/11	not estimable	0
Erdem 1993	6/20	9/24	0.80 [0.34, 1.86]	22.0%
Haque 1988	1/30	6/30	0.17 [0.02, 1.30]	16.1%
Samatha 1997	5/30	8/30	0.62 [0.23, 1.69]	21.5%
Shenoi 1999	7/25	7/25	1.00 [0.41, 2.43]	18.8%
Sidiropoulos 1981	4/41	8/41	0.50 [0.16, 1.53]	21.5%
**Total**	**23/157**	**38/161**	**0.63 [0.40, 1.00]**	**100%**

Review: IVIG in neonatal infection

Comparison: IVIG vs placebo or no intervention for suspected infection

Outcome: Mortality from any cause

**Table 2:** Mortality in trials of IVIG for proven infection in neonates

**Study**	**Exptl n/N**	**Ctrl n/N**	**Relative Risk (95% CI Fixed)**	**Weight**
Chen 1996	2/28	1/28	2.00 [0.19, 20.82]	3.8%
Erdem 1993	5/15	7/16	0.76 [0.31, 1.89]	25.4%
Haque 1988	1/21	4/23	0.27 [0.03, 2.26]	14.3%
Mancilla-Ramirez 1992	2/19	2/18	0.95 [0.15, 6.03]	7.7%
Samatha 1997	0/12	4/16	0.15 [0.01, 2.46]	14.6%
Sidiropoulos 1981	2/20	4/15	0.38 [0.08, 1.78]	17.2%
Weisman 1992	2/14	5/17	0.49 [0.11, 2.13]	17.0%
**Total**	**14/129**	**27/133**	**0.55 [0.31, 0.98]**	**100%**

Review: IVIG in neonatal infection

Comparison: IVIG vs placebo or no intervention for proven infection

Outcome: Mortality from any cause

The reviewers concluded that: *"The reduced mortality following treatment with IVIG for subsequently proven infection, the imprecise estimate of the effect size (number needed to treat 11, 95% CI 5.6, 100) and the borderline statistical significance for the outcome of mortality in neonates with suspected infection justify further research. Researchers should be encouraged to undertake well-designed trials to confirm or refute the effectiveness of IVIG" *[35].

Using slightly different selection criteria and methods for analysis, Jenson and Pollock have published a systematic review of three RCTs of IVIG in neonatal sepsis in which 55 infants received IVIG and 55 received placebo or no intervention [44]. The odds ratio for mortality in treated versus control infants was 0.173 (95% CI 0.03, 0.75). These authors reached a conclusion which many would consider premature, namely that "*IVIG should be considered as part of the routine therapy of neonatal sepsis*". However, it remains true that, among all the interventions currently reviewed in the Cochrane Library, IVIG therapy in neonatal sepsis is associated with one of the largest reductions in the odds of death. A further RCT of IVIG in neonatal sepsis in Brazilian neonatal units is being conducted. One of the applicants (K Haque) is an investigator of this trial. Its results will be incorporated into the current meta-analysis as soon as they are available.

Another Cochrane systematic review and meta-analysis of intravenous immunoglobulin used for treating sepsis and septic-shock in all patients (adults, children and neonates) suggested a beneficial effect of non-specific IVIG on all cause mortality (RR 0.64, 95% CI 0.51, 0.80) (Table 3) [45].

**Table 3:** Mortality in trials of IVIG for proven sepsis & septic shock in adults and children

**Study**	**Exptl n/N**	**Ctrl n/N**	**Relative Risk (95% CI Fixed)**	**Weight**
Standard IVIG vs placebo or no intervention, ACM				
Chen 1996	2/28	1/28	2.00 [0.19, 20.82]	0.9%
De Simone 1988	7/12	9/12	0.78 [0.44, 1.39]	8.5%
Dominioni 1991	11/29	22/33	0.57 [0.34, 0.96]	19.3%
Grundmann 1988	15/24	19/22	0.72 [0.51, 1.03]	18.6%
Just 1986	6/13	9/16	0.82 [0.40, 1.70]	7.6%
Shenoi 1999	7/25	7/25	1.00 [0.41, 2.43]	6.6%
Weisman 1992	2/14	5/17	0.49 [0.11, 2.13]	4.2%
Subtotal	50/145	72/153	0.73 [0.57, 0.93]	65.8%
				
IgM-enriched IVIG vs placebo or no intervention, ACM				
Erdem 1993	6/20	9/24	0.80 [0.34, 1.86]	7.7%
Haque 1988	1/30	6/30	0.17 [0.02, 1.30]	5.6%
Schedel 1991	2/27	9/28	0.23 [0.05, 0.97]	8.3%
Wesoly 1990	8/18	13/17	0.58 [0.33, 1.04]	12.6%
Subtotal	17/95	37/99	0.48 [0.30, 0.76]	34.2%
**Total**	**67/240**	**109/252**	**0.64 [0.51, 0.80]**	**100%**

Review: Intravenous immunoglobulin for treating sepsis and septic shock

Comparison: Polyclonal IVIG vs placebo or no intervention

Outcome: All-cause mortality (ACM)

A sensitivity analysis was also conducted, including only seven trials of good and fair quality. This also suggested a decreased risk of mortality (RR 0.55, 95% CI 0.40, 0.79).

The same Cochrane review also explored the effect of monoclonal antibodies; the relative risks for both anti-endotoxins and anti-cytokines were similar and were of borderline statistical significance (anti-endotoxins RR 0.93, 95% CI 0.85, 1.02; anti-cytokines RR 0.92, 95% CI 0.86, 0.99). The authors concluded that although there was evidence that non-specific IVIG appears to be beneficial "*large, multi-centre studies are needed to confirm the effectiveness of polyclonal IVIGs in reducing mortality in patients with sepsis. These are particularly indicated for neonatal sepsis, where evidence for benefit is still conflicting*".

### Safety: No evidence of transmission of blood borne viruses or prion disease

The risk of transmissible infection by blood products remains a potent source of anxiety for many clinicians and patients. However, IVIG produced to modern standards of quality control is one of the safest blood products available. There have been no reports of transmission of viruses or prions by the IVIG to be used in this study.

Methods of production including ethanol fractionation, and the use of the pH4/pepsin virus inactivation procedure, in addition to the use of plasma originating from non-UK donors have reduced the risk of transmission of infection to an absolute minimum [46,47].

In particular, for prion disease, leucocytes represent the main source of infectivity in Creutzfeld-Jacob disease. Owing to the physico-chemical characteristics of the abnormal prion protein, the process of partitioning and filtration during fractionation further reduces the risk of transmission in IVIG [48]. This theoretical risk must be considered in the context of the significantly increased risk of mortality and morbidity in infants eligible for the study.

As a further safeguard, fractionation pools of IVIG are tested with PCR (polymerase chain reaction) for known blood borne viruses.

### Safety: No evidence of haemolysis related to T activation of red cells

Bacteria such as Clostridia can strip neuraminic acid residues from the red cell membrane, exposing the T antigen (T activation). Adult plasma contains anti-T antibodies, so transfusing newborn infants whose red cells are T activated with whole blood, unwashed red cells or unselected plasma may lead to polyagglutination and haemolysis [49]. However, anti-T antibodies are predominantly IgM immunoglobulins a fraction which is removed from the IVIG used in this study [50]. T activation is not a contra-indication to its use in neonatal sepsis.

Although neonatal haemolysis has been noted in association with IVIG, it was not clinically significant [51]. The UK Committee on Safety of Medicines has received no reports of neonatal haemolysis or other adverse reactions in association with IVIG over a 30 year period until the present (personal communication, September 1999).

### Current practice

IVIG is not currently widely used for prophylaxis or treatment of neonatal sepsis in UK NICUs. In 1997, a postal survey of all paediatricians who were members of the British Association of Perinatal Medicine was undertaken into practice in the investigation and treatment of neonatal sepsis [52]. Of the 181 (66%) who responded, only 13 (7%) used IVIG routinely as adjuvant therapy alongside antibiotics.

### Summary

There is good preliminary evidence that IVIG therapy may reduce mortality in severe neonatal sepsis. However, there is no information on longer term quality of survival, the number of babies included in the existing systematic reviews is small and the effect size seems larger than would be anticipated. As a consequence a reliable multicentre trial is needed to provide definitive evidence that IVIG therapy for severe neonatal sepsis is or is not of benefit, with mortality or major morbidity as the outcome. IVIG is not yet widely used as routine therapy. There remains, therefore, a window of opportunity to perform such a trial before an intervention which has been inadequately assessed begins to be incorporated into routine practice.

## Methods and design

### Trial Eligibility

Hospitals will be eligible for entry if they can provide neonatal intensive or special care, can achieve satisfactory rates of follow up at two years and would be able to institute the routine use of adjuvant IVIG for babies with sepsis if the trial demonstrates evidence of benefit.

Babies are eligible if:

1. They are receiving antibiotics and have proven or suspected serious infection

AND

2. They have at least one of following:

• birthweight less than 1500 g OR

• evidence of infection in blood culture, CSF or usually sterile body fluid OR

• respiratory support via an endotracheal tube

AND

3. There is substantial uncertainty that IVIG is indicated

Exclusion criteria are:

1. IVIG has already been given

2. IVIG is thought to be needed or contra-indicated (e.g. because of severe congenital abnormality or contra-indications in the manufacturer's licensed product information sheet)

### Recruitment and Trial Entry

Recruitment will depend on good teamwork, knowledge and confidence among all clinicians, particularly front line nursing and medical staff, so that parents receive appropriate information about the study before entry and throughout their baby's stay. The ORACLE study recruited over 11,000 infants in 161 centres [53,54]. Experience from that trial suggests that it is helpful if nurses and doctors understand the study background, see clinical research as an integral part of neonatal care contributing to future quality of care, and if a named nurse is appointed and trained as a local Trial Co-ordinator. If those caring for the baby are well informed about the study, they can discuss it without transmitting anxiety. Indeed, parents are likely to feel less anxious if given the opportunity to discuss the options for their baby's treatment in the context of the study with knowledgeable staff.

The named nursing and medical representative in each unit will therefore receive opportunities for training, regular information and support to enable them to orientate and update new and established nursing and medical staff. The protocol, printed materials and relevant new research will be widely available and staff will be kept informed by newsletters, personal visits and the study website .

Many babies receive antibiotics for sepsis during their stay in a neonatal unit. Some babies may be given antibiotics more for prophylaxis than for treatment of suspected sepsis. The threshold for considering entry into INIS must be that there is a clinical suspicion that this baby has sepsis. Once an infant is considered eligible, it is important that enrolment takes place as soon as practically possible.

All parents should routinely be given an information leaflet about INIS by the nursing staff when their baby is admitted to the neonatal unit. This will include details of their local medical and nursing contact with whom they can discuss the study [55]. If their baby becomes eligible they will be asked for consent to participate in the study, and later follow up, by the most appropriate member of staff available, in person or by telephone. If they consent in person a copy of the signed consent form will be given to the parent(s). If telephone consent is considered necessary and appropriate by the recruiting clinician, a 'Telephone Consent' form will be completed. This form should then be read and signed by the parent(s) at their next visit to the hospital. Once this has happened, a copy of the consent form will be given to the parent(s).

### Treatment Allocation

The practical arrangements for random allocation to trial groups will be as simple as possible, based on that used in the ORACLE study [53,54]. Staff will open the next sequentially numbered study pack kept in the neonatal unit, which contains all the materials necessary to give a course of study drug.

### Clinical Management

#### IVIG group

an intravenous infusion of IVIG of 10 ml per kg, repeated after 48 hours.

#### Control group

an intravenous infusion of 10 ml per kg of 0.2% albumin solution (placebo) repeated after 48 hours.

Both infusions are of identical appearance: they are colourless and froth on agitation [see Additional File 1].

### Administration of treatment

The IVIG or placebo infusion will be given according to the manufacturer's instructions, over about 4–6 hours. A second, similar, dose will be given at or around 48 hours after the first dose. No further IVIG or placebo should be given, in this or any subsequent episodes of sepsis.

### Neonatal management

All other aspects of neonatal management will be left to the discretion of the paediatrician responsible for care. No special investigations and no delays of discharge will be required.

### Measurement of Outcome

#### Primary outcome measure

1. Mortality or major disability at two years, corrected for gestational age

#### Secondary short term outcomes

2. Mortality, chronic lung disease or major cerebral abnormality before hospital discharge, significant positive culture after trial entry, pneumonia, necrotising enterocolitis, duration of respiratory support

#### Secondary long term outcomes

3. Mortality before two years, major disability at 2 years, non-major disability at 2 years

#### Health service utilisation

4. Length of hospital stay and number of hospital admissions

### Data Collection

Hospital mortality, chronic lung disease, major cerebral abnormality and length of stay will be assessed from case notes. Major disability at two years will be assessed by questionnaires sent to the child's parents and health care professionals. Major disability will be defined according to the criteria set out in the National Perinatal Epidemiology Unit (NPEU) and Oxford Regional Health Authority document and will include any major disability in the following domains: neuromotor function, seizures, auditory function, communication, visual function, cognitive function and other physical disability [56,57].

The parental questionnaire incorporates the parent report component of the Parent Report of Children's Abilities (PARCA), which was used in the 2 year follow up of the MRC funded UKOS trial [58]. This shortened version of the PARCA was acceptable to parents, with a high response rate in UKOS, and is currently being validated by the UKOS team. The PARCA score (both parent report and parent administered components) has been validated and was found to predict performance on the Mental Development Index of the Bayley Scales of Infant Development II [58,59]. The overall score for the modified PARCA will give a measure of verbal and non-verbal cognitive abilities. The parents' questionnaire also includes questions about temperament, which may give an early indication of behavioural and attentional difficulties, and also includes questions about respiratory function, hearing, vision, hospital admissions, relevant diagnoses and current function in a number of domains, allowing categorisation of disability as major or non-major.

Experience of other trials in this area at the NPEU, Oxford and elsewhere, suggests that it is possible to determine early neonatal events for all babies recruited. Loss to follow-up after hospital discharge of the child is more problematic.

There are likely to be few children who cannot be traced in the UK either through the hospital of recruitment or the NHS Central Register. Similar high rates of follow-up will be expected in countries participating outside the UK.

### Analysis

An intention to treat analysis will be performed comparing the outcome of all children allocated IVIG with all those allocated placebo, regardless of what treatment was received, or how complete that treatment was. Statistical analysis will calculate the relative risk of an outcome in the IVIG group compared with the placebo group along with a 95% confidence interval. For subgroup analyses, 99% confidence intervals will be calculated to take account of the number of comparisons.

#### Subgroup analyses

Ten subgroup analyses will also be undertaken, stratifying by the factors described below.

1. Birth weight. Infants of very low birth weight (VLBW: < 1500 g) v infants with birth weight ≥ 1500 g

2. Small for gestational age infants (< 10^th ^centile) v infants ≥ 10^th ^centile

3. Gestational age at birth: < 26 weeks, 26+0 to 27+6 weeks, 28+0 to 29+6 weeks, 30+0 weeks or more

4. Gender: male vs female

5. Maternal chorioamnionitis: infants born at < 30 weeks gestation to women with clinical chorioamnionitis v infants born at < 30 weeks gestation with no clinical chorioamnionitis v infants born at ≥ 30 weeks

6. Elevated maternal CRP: infants born at < 30 weeks gestation to women with elevated CRP (> 80 mg/l) v infants born at < 30 weeks gestation with no elevated maternal CRP v infants born at ≥ 30 weeks

7. Preterm birth and duration of membrane rupture: Born at < 37 weeks and membranes ruptured for < 24 hours, 24–48 hours or > 48 hours versus born at ≥ 37 weeks

8. Clinical markers of mortality risk:

(i) Clinical evidence of high mortality risk: looking seriously ill or inactive, and has:

(a) capillary refill time > 3 seconds OR

(b) bowel perforation or definite necrotising enterocolitis OR

(c) prolonged bleeding from puncture sites OR

(d) ventilated, SaO_2_/FiO_2 _ratio or PaO_2_/FiO_2 _ratio consistent with > 15% mortality risk for gestation OR

(e) pH consistent with > 15% mortality risk for gestation. [SaO_2_/FiO_2 _ratio, PaO_2_/FiO_2 _ratio and pH consistent with > 15% mortality risk will be extrapolated from oxygenation and pH data in a prospective cohort of 14,000 infants (UK Neonatal Staffing Study) by methods similar to that used in the development of the MRC funded CRIB score [6]]

(ii) Intermediate mortality risk: not satisfying criteria for high risk, but has:

(a) Total white cell count < 5 × 10^9^/l OR

(b) CRP above 15 mg/l OR

(c) platelet count < 50 × 10^9^/l OR

(d) organism(s) isolated in blood or usually sterile site OR

(e) pneumonia on chest X-ray OR

(f) CSF consistent with bacterial meningitis

(iii) Other: not satisfying criteria for high or intermediate risk

9. Type of infection:

(i) Early onset infection (non-contaminant organisms isolated from culture sent before 48 hours):

(a) group B streptococcal disease

(b) other pathogens

(c) indeterminate aetiology

(ii) Late onset infection (non-contaminant organism isolated from culture sent after 48 hours):

(a) gram positive organisms except *coagulase negative staphylococcus*

(b) *coagulase negative staphylococcus*

(c) gram negative organisms

(d) fungal infection

(e) other pathogens

(f) indeterminate aetiology

(iii) Post surgery

10. Type of IVIG. This subgroup analysis will analyse separately babies recruited in hospitals using the different IVIG products included in INIS. This subgroup analysis will include baseline characteristics and treatments after randomisation as well as outcomes.

### Interim analyses: the Data Monitoring and Ethics Committee

For the trial, a Data Monitoring and Ethics Committee (DMEC) has been established. This is independent of the trial organisers and meets at least once per year. During the period of recruitment to the trial, interim analyses are supplied, in strict confidence, to the DMEC, together with any other analyses the DMEC may request. In the light of interim data, and other evidence from relevant studies (including updated overviews of the relevant randomised controlled trials), the DMEC will inform the Trial Steering Committee, if in their view: i) there is proof beyond reasonable doubt that the data indicate that any part of the protocol under investigation is either clearly indicated or contra-indicated, either for all infants or for a particular subgroup of trial participants, or ii) it is evident that no clear outcome will be obtained. Decision to inform the Trial Steering Committee in either of these circumstances will in part be based on statistical considerations.

Appropriate criteria for proof beyond reasonable doubt cannot be specified precisely. A difference of at least 3 standard deviations in the interim analysis of a major endpoint may be needed to justify halting, or modifying, such a study prematurely. If this criterion were to be adopted, it would have the practical advantage that the exact number of interim analyses would be of little importance, and so no fixed schedule is proposed [60].

Unless modification or cessation of the protocol is recommended by the DMEC, the Trial Steering Committee, collaborators and administrative staff (except those who supply the confidential information) will remain ignorant of the results of the interim analysis. Collaborators and all others associated with the study may write through the trial office to the DMEC to draw attention to any concern they may have about the possibility of harm arising from the treatment under study, or about any other matters that may be relevant.

The membership of the Data Monitoring and Ethics Committee is :

Professor Adrian Grant (Chair)

Professor Forrester Cockburn

Professor Deborah Ashby

Mrs Hazel Thornton

Professor Neena Modi

Dr Brian McClelland

### Sample Size and Feasibility

Table 4 shows positive blood culture rates (including probable contaminants) in 3,963 consecutive infants of all birthweights admitted to a randomly selected, nationally representative cohort of 54 UK neonatal units between 1st March 1998 and 4th September 1998 in a study of organisation and outcomes of neonatal care funded by the NHS Executive [6].

**Table 4:** Positive blood culture rates

**Number with positive cultures**	**Mortality (all causes)**	**Number with negative cultures**	**Mortality (all causes)**
204/3,963 (5%)	16/204 (8%)	3,759/3,963 (95%)	95/3,759 (2.5%)

Among VLBW infants with positive blood cultures, mortality was 14%. In a North American cohort, mortality in VLBW infants with septicaemia was 21% [7]. Assuming combined rates of mortality and major morbidity of 10–20% for all infants and 20–30% for VLBW infants, Table 5 outlines estimated sample sizes.

**Table 5:** Range of estimated sample sizes

**Mortality or major morbidity in control group**	**Mortality or major morbidity in IVIG group**	**Relative risk reduction**	**Total sample size required to detect difference with 95% confidence**	
			
			**80% power**	**90% power**
30%	26%	13%	4,052	5,392
30%	25%	17%	2,580	3,428
30%	20%	33%	626	824
25%	21%	16%	3,572	4,748
25%	20%	20%	2,266	3,006
25%	15%	40%	540	708
20%	16%	20%	2,994	3,972
20%	15%	25%	1,890	2,502
20%	12.5%	37.5%	810	1,066
15%	12%	20%	4,204	5,582
15%	10%	33%	1,450	1,914
12%	9%	25%	3,408	4,516
10%	7.5%	25%	4,166	5,524

### Feasibility

About 5,000 infants will be needed to demonstrate moderate reductions in mortality or survival with major developmental delay with adequate power. Over a three year recruitment period, assuming that 7–10% of all admissions are diagnosed with clinical sepsis and considered eligible for recruitment, 150 NICUs with an average of 300 admissions per year will be required to achieve the recruitment target, assuming a 40–50% rate of recruitment of eligible infants. Neonatal units will initially be recruited in the UK, Australia and New Zealand. A census of all 186 UK neonatal intensive care units (NICUs) and 60 special care baby units in 1996 (which is a 100% response rate) found that the median number of admissions per year per NICU was 317 [61]. If a broadly representative sample of about half of all UK NICUs and SCBUs participate, then over 50% of the projected recruitment rate for the trial will be possible within the UK, leaving the additional 50% to be recruited from the rest of the world.

This study reflects the philosophy that the only practicable way to achieve comparisons which are sufficiently large to minimise the risk of being seriously misled by the play of chance is to design trials that are extremely simple and flexible [62].

Experience in the OSIRIS and ORACLE studies suggests that a large, simple trial of this scale of a potentially important intervention is feasible [63,53,54]. Furthermore, systematic reviews of RCTs of IVIG therapy in neonatal sepsis suggest a substantial reduction in mortality. This contrasts with the systematic reviews of RCTs of antibiotics in threatened preterm birth which led to the ORACLE study, as these showed no evidence of a difference in neonatal mortality. This preliminary evidence that IVIG may reduce mortality may further enhance the appeal of the study.

The estimate of the incidence of the outcome (the event rate) for the trial is imprecise, particularly as the threshold at which clinicians will enter patients cannot be estimated. If clinicians enter babies where the likelihood of serious sepsis is lower then the event rate will also be lower. If clinicians restrict entry to only those babies who are very sick, then the event rate will be high. Either of these two scenarios is reasonable because it will define a population to which the trial result can be generalised. However, it does mean that until the trial has recruited sufficient numbers of babies it will not be possible to determine the optimum trial sample size with any certainty. As a consequence the trial sample size currently represents the minimum size desirable. Assuming the trial recruits for three years, the maximum number of babies which can be recruited during this time will be recruited and it is possible that this number may exceed 5,000. During recruitment to the trial the accumulating data will be seen by an independent Data Monitoring and Ethics Committee at least once per year (see above) and they will advise the Trial Steering Committee whether the trial has answered the clinical question being addressed. If not, the trial will continue to recruit until 5,000 babies have been recruited, or until funding is exhausted.

### Publication Policy

To safeguard the scientific integrity of the trial, data from this study should not be presented in public or submitted for publication without requesting consent from the Trial Steering Committee (see Organisation below). The success of the trial depends on the collaboration of a large number of doctors and nurses. For this reason, chief credit for the results will be given not to the committees or central organisers but to all who have collaborated in the study. Acknowledgement will include all members of the trial committees, the data co-ordinating centre, trial staff, and local co-ordinators at all collaborating centres. Authorship at the head of the paper will take the form; "The INIS Study Collaborative Group". This is the preferred option, as it avoids giving undue prominence to any individuals. All contributors to the study will be listed at the end of the report, with their contribution to the study identified.

## Organisation

The protocol received Multi-Centre Research Ethics Committee (MREC) approval from the Anglia and Oxford MREC on 16 October 2000 (Reference number: 00/05/53). Each country will obtain ethics approval prior to recruitment commencing.

### Trial Steering Committee (see Table 6)

The Trial Steering Committee (TSC) provides overall supervision of the trial on behalf of the Medical Research Council. Its terms of reference are:

1. To monitor and supervise the progress of the trial towards its interim and overall objectives

2. To review at regular intervals relevant information from other sources (e.g. related trials)

3. To consider the recommendations of the Data Monitoring and Ethics Committee

4. In the light of 1, 2 and 3 above, to inform the MRC Council and relevant MRC Research Boards on the progress of the trial

5. To advise the MRC Council on publicity and the presentation of all aspects of the trial

**Table 6:** The membership of the Trial Steering Committee is:

Professor Richard Cooke	Professor of Neonatal Medicine	Chair
Dr Tim Neal	Consultant in Medical Microbiology	Independent member
Dr Gorm Greisen	Consultant Neonatologist	Independent member
Professor Douglas G Altman	Statistician	Independent member
Farrah Pradhan	BLISS representative	Independent member
Dr William Tarnow-Mordi	Consultant Neonatologist	Co-Investigator
Jana Voigt	Programme Manager	MRC representative
Professor Peter Brocklehurst	Perinatal Epidemiologist	Chief Investigator

Meetings of the TSC will take place at least once per year.

### Study Investigators' Group

The Investigators' Group will consist of the trial investigators, representatives of specific groups whose expertise is necessary for the trial, and investigators of any ancillary studies. This group will supervise the practical aspects of the trial's conduct. It will resolve problems brought to it by the Project Management Group (see below) and will be responsible for organising reporting and dissemination of the trial's results.

### Project Management Group

The Project Management Group will oversee the day-to-day running of the trial.

The responsibilities of the Project Management Group include:

i) Recruitment of participating centres

ii) Distribution and supply of data collection forms and other appropriate documentation for the trial

iii) Data collection and management

iv) Organisation of the distribution system for the treatment packs

v) Organisation of the follow-up of children at 2 years of age, including the distribution of questionnaires, follow-up of non-responders and liaison with local 'follow-up' personnel

vi) Data entry and cleaning

vii) Data analysis

viii) Collection of adverse event data

ix) Organising and servicing the Data Monitoring and Ethics Committee

### Local Co-ordination

Each participating centre will identify a local medical co-ordinator and a local neonatal nurse co-ordinator (as necessary). The responsibility of the local co-ordinators will be to:

i) Be familiar with the trial

ii) Liaise with the INIS Co-ordinating Centre

iii) Ensure that all staff involved in the care of babies on the neonatal unit are informed about the trial

iv) Ensure that mechanisms for recruitment of eligible babies (including information material) are in place, monitor their effectiveness, and discuss reasons for the non-recruitment of any eligible babies with relevant staff

v) Ensure that supplies of data collection forms are always available, that they are completed and returned to the INIS Co-ordinating Centre promptly, and to deal with any queries arising

vi) Notify the INIS Co-ordinating Centre of any serious adverse events

vii) Facilitate other aspects of local collaboration as appropriate

viii) Make all data available for verification, audit and inspection purposes as necessary

ix) Ensure that the confidentiality of all information about trial participants is respected by all persons

## Competing interests

The authors declare that they have no competing interests.

## Authors' contributions

Members of the INIS Study Investigators' Group, representing the INIS Study Collaborators were involved in the conception and design of the study.

PB of the National Perinatal Epidemiology Unit, Oxford, drafted the manuscript. All members of the INIS Study Investigators' Group edited the manuscript, read and approved the final protocol.

Members of the INIS Study Investigators' Group are :

Professor Peter Brocklehurst - NPEU, Oxford

Mrs Sally Brearley - Sutton, Surrey

Dr Khalid Haque - St Helier Hospital, Surrey

Mr Andy Leslie - City Hospital, Nottingham

Dr Alison Salt - Moorfields Eye Hospital, London

Dr Ben Stenson - Royal Infirmary of Edinburgh, Edinburgh

Mr Jim Stephenson - St Helier Hospital, Surrey

Professor William Tarnow-Mordi - Westmead Hospital, Sydney, Australia

## Pre-publication history

The pre-publication history for this paper can be accessed here:



## Supplementary Material

Additional file 1 **Contents of IVIG and placebo upon reconstitution with 60 ml water for injection.** The data provided show the contents IVIG and placebo upon reconstitution with 60 ml water for injection. Click here for file
