# Supplementary material for: The INIS Study. International Neonatal Immunotherapy Study: non-specific intravenous immunoglobulin therapy for suspected or proven neonatal sepsis: an international, placebo controlled, multicentre randomised trial
Source: BMC Pregnancy Childbirth. 2008 Dec 8;8:52. doi: 10.1186/1471-2393-8-52 (PMC2626572; doi:10.1186/1471-2393-8-52)
Supplement: Additional file 1 — Contents of IVIG and placebo upon reconstitution with 60 ml water for injection. The data provided show the contents IVIG and placebo upon reconstitution with 60 ml water for injection. [file 1471-2393-8-52-S1.doc]

**Additional File 1**

**Contents of IVIG and Placebo upon reconstitution with 60ml Water for Injection**

|  | **IVIG*** | **Placebo** |
| --- | --- | --- |
| Total Protein (g/L) | 44.5±0.8 | 2.0±0.5 |
| Sodium (mMol) | 44±2 | 48 |
| Potassium (mMol) | 0.15 | 0.17 |
| pH | 6.9±0.1 | 6.65 |
| Osmolality (mOsm/kg) | 403±11 | 413 |
| Ethanol (ml/L) | <0.1 | <0.1 |
| Citrate (mMol) | <0.1 | <0.1 |
| Albumin (g/L) | <0.1 | 2.0±0.5 |
| Sucrose (%) | 9.0±0.3 | 10.5 |
| HPLC^#^ (% Aggregate) | 2.3±0.3 | 4.3 |
| PKA ^ (iu/ml) | <2.0 | <2.0 |

* mean ± SD of 20 batches of 3g IVIG

^#^ high purity liquid chromatography

^ pre-kallirien activator
